# Response of the Unicellular Diazotrophic Cyanobacterium *Crocosphaera watsonii* to Iron Limitation

**DOI:** 10.1371/journal.pone.0086749

**Published:** 2014-01-21

**Authors:** Violaine Jacq, Céline Ridame, Stéphane L'Helguen, Fanny Kaczmar, Alain Saliot

**Affiliations:** 1 Université Pierre et Marie Curie, UMR LOCEAN -IPSL/CNRS/IRD/MNHN, Paris, France; 2 Université de Brest, CNRS/IRD, UMR 6539, LEMAR, OSU-IUEM, Plouzané, France; Mount Allison University, Canada

## Abstract

Iron (Fe) is widely suspected as a key controlling factor of N_2_ fixation due to the high Fe content of nitrogenase and photosynthetic enzymes complex, and to its low concentrations in oceanic surface seawaters. The influence of Fe limitation on the recently discovered unicellular diazotrophic cyanobacteria (UCYN) is poorly understood despite their biogeochemical importance in the carbon and nitrogen cycles. To address this knowledge gap, we conducted culture experiments on *Crocosphaera watsonii* WH8501 growing under a range of dissolved Fe concentrations (from 3.3 to 403 nM). Overall, severe Fe limitation led to significant decreases in growth rate (2.6-fold), C, N and chlorophyll *a* contents per cell (up to 4.1-fold), N_2_ and CO_2_ fixation rates per cell (17- and 7-fold) as well as biovolume (2.2-fold). We highlighted a two phased response depending on the degree of limitation: (i) under a moderate Fe limitation, the biovolume of *C. watsonii* was strongly reduced, allowing the cells to keep sufficient energy to maintain an optimal growth, volume-normalized contents and N_2_ and CO_2_ fixation rates; (ii) with increasing Fe deprivation, biovolume remained unchanged but the entire cell metabolism was affected, as shown by a strong decrease in the growth rate, volume-normalized contents and N_2_ and CO_2_ fixation rates. The half-saturation constant for growth of *C. watsonii* with respect to Fe is twice as low as that of the filamentous *Trichodesmium* indicating a better adaptation of *C. watsonii* to poor Fe environments than filamentous diazotrophs. The physiological response of *C. watsonii* to Fe limitation was different from that previously shown on the UCYN *Cyanothece* sp, suggesting potential differences in Fe requirements and/or Fe acquisition within the UCYN community. These results contribute to a better understanding of how Fe bioavailability can control the activity of UCYN and explain the biogeography of diverse N_2_ fixers in ocean.

## Introduction

In oligotrophic oceanic regions, bioavailable nitrogen (N) concentrations are sufficiently low that they set a constraint on primary productivity [1]. Diazotrophic cyanobacteria are not affected by N limitation due to their ability to use the dinitrogen (N_2_) dissolved in oceanic surface waters as an alternative source of N. As N_2_ represents an effectively unlimited resource of N, the N_2_ fixation ability confers a major ecological advantage to diazotrophic cyanobacteria relative to non-diazotrophic phytoplankton in N depleted tropical and subtropical waters [2]. On a global scale, N_2_ fixation represents the largest source of newly-fixed N to the open ocean (120 TgN yr^−1^ [3]), supporting a part of new primary production and influencing the N and carbon (C) cycles [4], [5]. In the tropical and subtropical North Atlantic and Pacific oceans, N_2_ fixation is estimated to support up to half of the new and export production [5], [6], playing a key role in the uptake of atmospheric CO_2_ by increasing the strength of the biological pump.

Among environmental factors constraining the distribution of diazotrophic cyanobacteria and the magnitude of N_2_ fixation, iron (Fe) is widely believed to be a key controlling factor as the nitrogenase enzyme complex involved in intracellular N_2_ reduction in NH_3_ is Fe rich [7]. Furthermore, the high energetic cost of N_2_ fixation imposes an additional Fe requirement for increase photosynthetic capacity [8], [9]. The extremely low Fe solubility in oxic seawater [10] led to dissolved Fe (dFe) concentrations lower than about 1 nM in the open surface ocean [11]–[13], resulting in the potential limiting role of Fe for marine diazotrophic cyanobacteria. The effects of Fe limitation on the growth and N_2_ fixation of the filamentous marine diazotrophic cyanobacteria *Trichodesmium* sp. have been widely evidenced both in artificial [14]–[16] and natural [17] environments. *Trichodesmium* sp. had been assumed to be the dominant N_2_-fixing organism in the open ocean [18], [19] until the recent discovery of unicellular diazotrophic cyanobacteria (UCYN, including UCYN-A, -B and -C [20]). Field measurements have highlighted that N_2_ fixation rates associated with UCYN probably equal or exceed those associated with *Trichodesmium* sp. at regional scale [4]. N_2_ fixation rates associated with UCYN were estimated to be up to 75% of the total N_2_ fixation rate in the equatorial Western Pacific under stratified conditions [21]. On a global scale, recent biogeochemical models attribute about 50% of the total oceanic N_2_ fixation rate to unicellular analogues [22]. Despite the biogeochemical importance of UCYN, their controlling factors remain poorly known. To date, only one open ocean UCYN species is available in culture: *Crocosphaera watsonii* (UCYN-B). Culture-based and field experiments have shown that light [23], [24], temperature [24], [25] and phosphorus [26]–[29] can control the growth of *Crocosphaera*, but the effects of Fe limitation on UCYN have been poorly investigated. The few studies conducted on the impact of Fe limitation on *C. watsonii* highlighted notable change in expression of several proteins under Fe stress [30], [31]. Decreases in the N_2_ fixation and growth rates of *C. watsonii* have been observed in one Fe-limited culture [32] and recent field enrichment experiments in the tropical Atlantic and Pacific have revealed that abundance of UCYN-B could be Fe limited in their natural habitats [27], [28], [33]. The response of *C. watsonii* to Fe limitation remains not fully characterized and needs to be quantified. In order to improve our knowledge and understanding of the impact of Fe limitation on UCYN, we conducted trace-metal clean culture experiments of *C. watsonii* WH8501 cultivated under a range of dFe concentrations to quantify for the first time the impact of Fe limitation on the growth, N_2_ fixation rate, primary productivity, elemental contents, and cell size of an open ocean UCYN.

## Materials and Methods

### Culture experiments

All bottles and labware were thoroughly cleaned with suprapur HCl acid and ultra-pure water (>18.2 MΩ). All manipulations were conducted in a clean laboratory within a sterile laminar flow hood (class 100) using sterile and trace metal clean techniques. Batch cultures of *C. watsonii* WH8501 were grown in sterile polycarbonate bottles at 27.5°C, under a 12∶12 h light∶dark cycle at a light intensity of ∼150 µmol photons.m^−2^.s^−1^. The cells were cultivated in N free YBCII medium [34], prepared with Suprapur® quality salts and reagents and amended at different dFe concentrations. The medium contained phosphate (20 µM), vitamins (B_12_, thiamine and biotin) and trace metals (Co, Mo, Cu, Zn and Mn). It was sterilized by autoclaving and 0.2 µm filtration. Cultures were gently mixed using orbital shakers to minimize cell sedimentation. Fe (FeCl_3_) was added in triplicate cultures to obtain different final dFe concentrations ranging from 0 to 400 nmol.L^−1^ (nM) and was complexed with 2 µM of ethylenediaminetetra-acetic acid (EDTA), a metal ion buffering agent. In order to quantify a potential Fe contamination, dFe was analysed in sterilized YBCII medium before Fe addition by flow injection with online preconcentration and chemiluminescence detection [35] at the LOV laboratory (Villefranche sur mer). A background concentration of 3.3 nM was found in the medium and was systematically included in our results. Consequently, the eight dFe concentrations in the triplicate cultures were 3.3, 5.3, 8.3, 13.3, 23.3, 43.3, 103.3 and 403.3 nM (Table 1). Cells were previously acclimated to these different Fe concentrations for a minimum of 35 generations. Flow cytometry measurements (LOMIC laboratory) showed that our cultures were not axenic and allowed the determination of the abundance and biovolume of bacteria [36]. Using a conversion factor between biovolume and C content of bacteria from [37], we found that the C content associated with bacteria represented on average 0.4% of the total particulate organic carbon (POC) in the cultures. The initial pH in the cultures was 8.15 and variations between the beginning and the end of the growth phase were lower than 0.2 pH units, which avoided CO_2_ limitation and pH effects on Fe chelation by EDTA [38].

**Table 1:** Influence of dFe concentrations in the cultures on surface∶volume ratio, elemental ratios, cellular and volume-normalized N_2_ and CO_2_ fixation rates of *C. watsonii* WH8501 (numbers in brackets represent standard deviation).

Media dFe (nM)	3.3	5.3	8.3	13.3	23.3	43.3	103.3	403.3
Surface∶volume	3.1	ND	ND	2.9	ND	2.9	ND	2.4
(µm^2^∶µm^3^)	(0.2)			(0.2)		(0.1)		(0.3)
C∶N ratios	8.3	8.9	8.8	10.2	9.5	8.3	8.0	9.6
(mol∶mol)	(0.5)	(0.3)	(0.6)	(0.4)	(0.9)	(0.3)	(0.8)	(0.5)
Chl *a*∶C ratios	51.1	41.0	34.1	36.6	54.7	63.6	66.5	58.1
(µmol∶mol)	(8.5)	(3.6)	(7.7)	(4.2)	(6.6)	(6.8)	(8.4)	(5.3)
N_2_ fixation	7.2	ND	ND	ND	ND	53.2	ND	65.6
(amol N.µm^−3^.h^−1^)	(1.2)					(7.3)		(22.2)
CO_2_ fixation	1.2	ND	ND	3.1	ND	3.5	ND	3.9
(fmol C.µm^−3^.h^−1^)	(0.2)			(0.6)		(0.5)		(1.3)

ND : No data.

All the parameters discussed in this study, except cell abundance, were determined during the exponential growth phase. Our results are reported as a function of dFe concentrations as well as dissolved inorganic Fe concentrations, hereafter referred to as Fe′ and representing hydrolysed forms of dFe, supposed to be the bioavailable forms of Fe in EDTA buffered artificial seawater [39], [40]. Fe′ concentrations were computed from the Fe-EDTA complexation data in [38], taking into account influence of pH, light and temperature. The resulting estimated Fe′ concentrations in the media ranged from 0.16 to 20.16 nM. The 3 highest Fe′ concentrations (2.16, 5.16 and 20.16 nM) are invalid as they exceed the solubility limit for Fe with respect to ferric hydroxide precipitation, which is assumed to be ∼1.5 nM based on experimental data from [38].

### Cell abundance and growth rate

*C. watsonii's* abundance was monitored by daily cell counts with an epifluorescence microscope (Nikon Eclipse 50i) using natural fluorescence of chlorophyll *a* (Chl *a*). These data were highly similar to those obtained by flow cytometry measurements (data not shown). Specific growth rates in the exponential phase were determined from linear regression of the logarithmic transformed cell abundance versus time.

### Cell biovolume

*C. watsonii* cells were harvested in exponential phase, 2 hours after the beginning of the dark period, onto 0.4 µm polycarbonate membranes, and incubated overnight into a fixative with adjusted osmolarity (3% glutaraldehyde in 0.1M cacodylate pH 7.4, NaCl 1.75%). Membranes were then washed, post-fixed for 1 h with 1% osmium tetroxide in 0.1M cacodylate buffer with 1.75% NaCl, and then dehydrated with graded increasing concentrations of ethanol (50, 70, 96, 100%) and critical point dried (CPD 7501, Quorum Technologies). Finally, membranes were mounted on stubs, gold-sputtered (Scancoat Six, Edwards) and observed with a conventional SEM (Scanning Electron Microscope, Cambridge Stereoscan S260). Pictures were analysed with ImageJ software [41] in order to determine cell diameters and biovolumes. Due to experimental constraints, cell diameters and biovolumes were determined on four cultures (dFe = 3.3, 13.3, 43.3 and 403.3 nM).

### Chlorophyll a

Culture samples were gently filtered (pressure<200 mbar) onto 0.7 µm glass microfiber filters (GF/F, Whatman©). Then, the filters were stored at −25°C. After extraction in 90% acetone [42], fluorescence of Chl *a* was measured at 670 nm on a Hitaschi F-4500 spectrofluorometer. Cellular Chl *a* content was calculated using the cell abundance at the day of sampling.

### CO_2_ fixation rate, N_2_ fixation rate, C and N content

CO_2_ fixation rates were determined using the ^13^C-tracer addition method [43]. Seven hours after the beginning of the light period, subsamples of cultures (from 25 to 500 ml) were incubated during 3.5 h with a small addition of NaH^13^CO_3_ (99%, Eurisotop) in order to obtain a final enrichment of about 10 atom% excess. N_2_ fixation rates were determined using the ^15^N_2_ gas-tracer addition method [44]. Incubations for CO_2_ and N_2_ fixation were not performed simultaneously as *C. watsonii* perform a nocturnal N_2_ fixation in order to avoid the inhibitory effects of oxygen on nitrogenase due to photosynthesis [45]. Briefly, 2 hours after the onset of the dark period, ^15^N_2_ gas (98.3%, EURISOTOP) was added to sub-samples of cultures (from 45 to 630 ml) in polycarbonate bottles equipped with septum caps using a gas-tight syringe, and bottles were incubated for 3.5 hours. ^15^N_2_ tracer was added to obtain a final enrichment of the N_2_ pool of about 10 atom% excess. After ^13^C and ^15^N_2_-incubations, samples were filtered onto pre-combusted 25 mm GF/F filters and filters were stored at −25°C. Prior to analysis, filters were dried at 40°C for 48 h. Particulate organic carbon (POC) and nitrogen (PON) concentrations as well as ^13^C- and ^15^N-enrichments were quantified with a mass spectrometer (Delta plus, ThermoFisher Scientific, Bremen, Germany) coupled with an elemental analyser (Flash EA, ThermoFisher Scientific) via a type III-interface. Standard deviations were 0.009 µM and 0.004 µM for POC and PON, respectively and 0.0002 atom% and 0.0001 atom% for ^13^C enrichment and ^15^N enrichment, respectively. N_2_ fixation rates were calculated by isotope mass balanced as described by [44]. Cellular C and N contents as well as molar C∶N ratios were estimated using the POC and PON determined during the light period and the cell abundance measured at the day of sampling. Relative N_2_ fixation was calculated as the rates of N_2_ fixation in the different Fe treatments normalized by the mean rate in the Fe-replete treatment. Relative CO_2_ fixation rates were determined using the same calculation.

### Statistical analysis

After checking homoscedasticity using a Bartlett test, means were compared using a one-way ANOVA and a pairwise-t-test with the Holm method for *p*-value adjustment (α = 0.05). In the case of heterogeneity of the variances, the tests were performed on the log-transformed data. The statistical tests, the Monod non-linear regression and derived growth parameters (maximum growth rate and half saturation constant for growth) were calculated using R software.

## Results and Discussion

### 1. The global influence of Fe limitation

**Figure 1:**
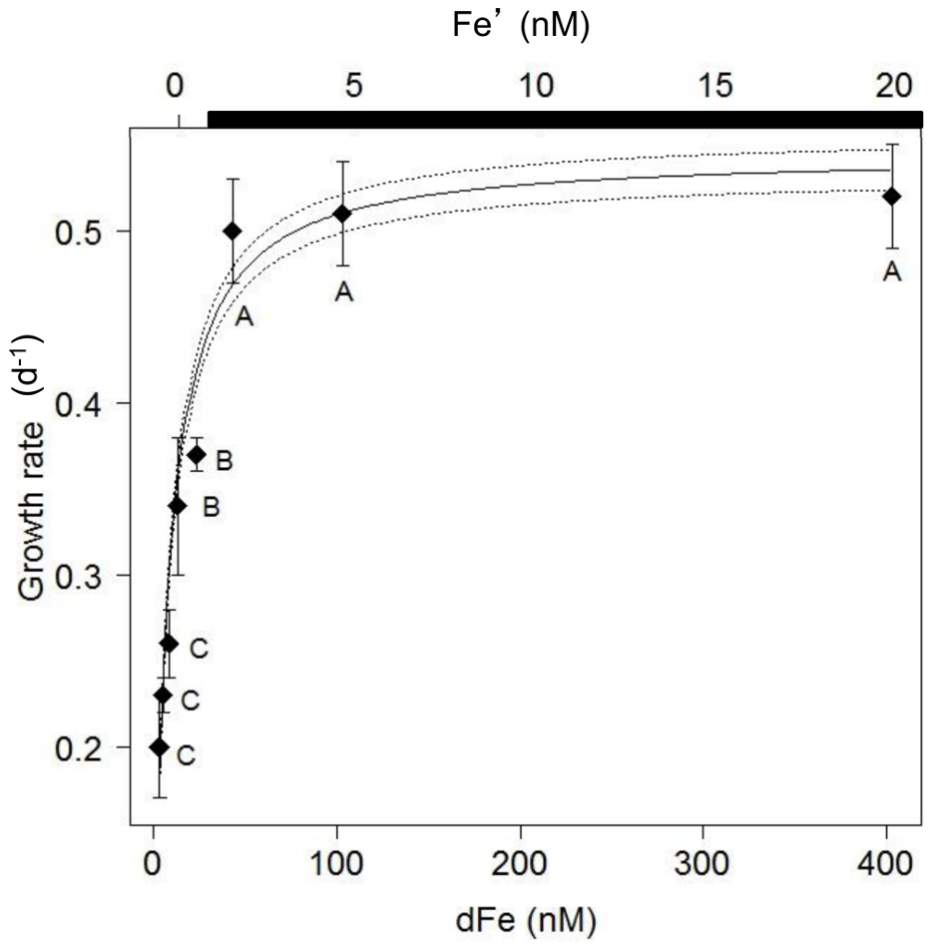
Growth rate of *C. watsonii* related to dFe and Fe′ concentrations. Error bars represent standard deviation; different letters correspond to statistically different means (*p*<0.05) and the black bar indicates the region of expected Fe hydroxide precipitation. The Monod regression, performed with dFe concentrations, is represented by the black line and standard deviation of regression by dotted lines.

**Figure 2:**
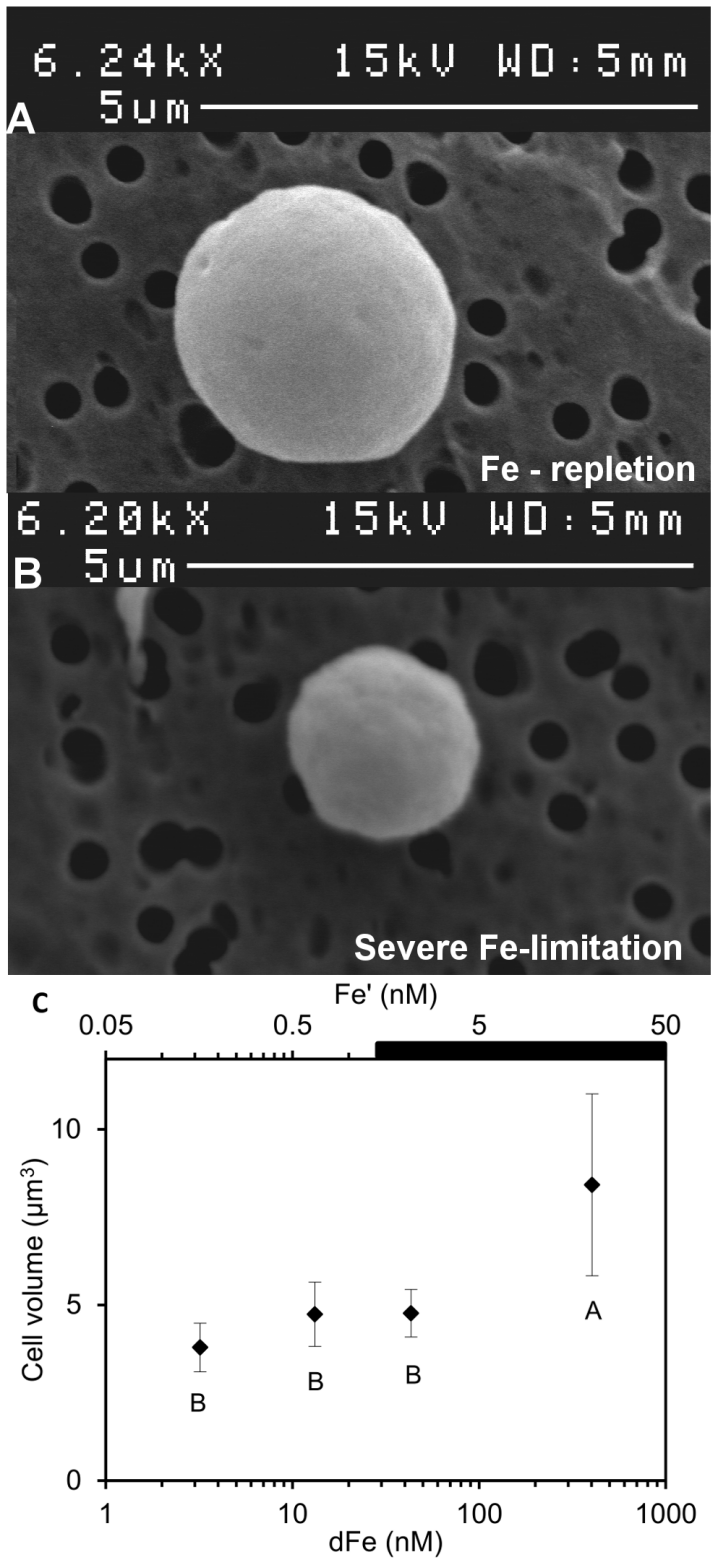
Influence of Fe availability on *C. watsonii* biovolume. Scanning electron microscopy photography of *C. watsonii* growing in (A) Fe-replete condition (dFe = 403.3 nM) and (B) severe Fe-limited condition (dFe = 3.3 nM). (C) Mean biovolume of *C. watsonii* related to dFe and Fe′ concentrations, in log scale. Error bars represent standard deviation; different letters correspond to statistically different means (*p*<0.05) and the black bar indicates the region of expected Fe hydroxide precipitation.

**Figure 3:**
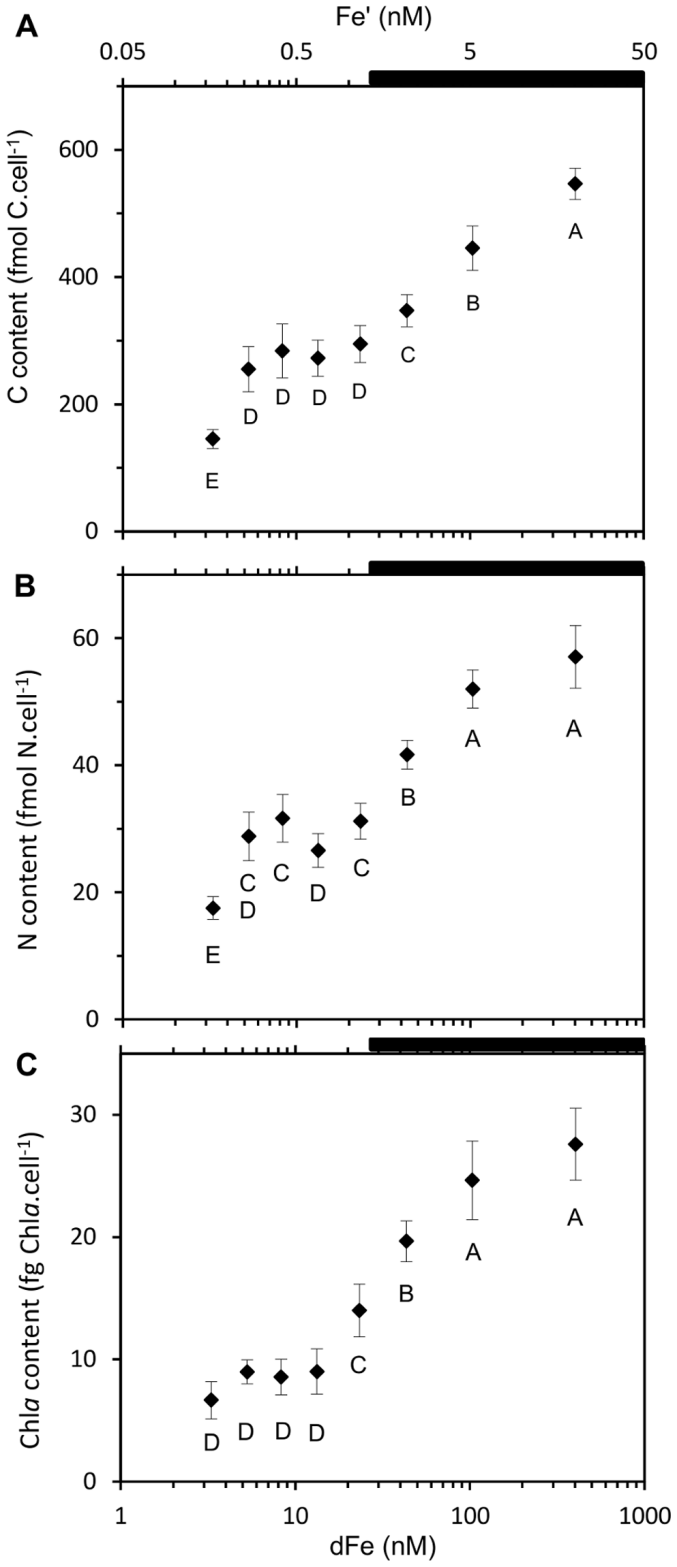
Influence of Fe availability on the elemental composition of *C. watsonii*. Mean cellular content of C (A), N (B) and Chl *a* (C) related to dFe and Fe′ concentrations, in log scale. Error bars represent standard deviation; different letters correspond to statistically different means (*p*<0.05) and the black bar indicates the region of expected Fe hydroxide precipitation.

**Figure 4:**
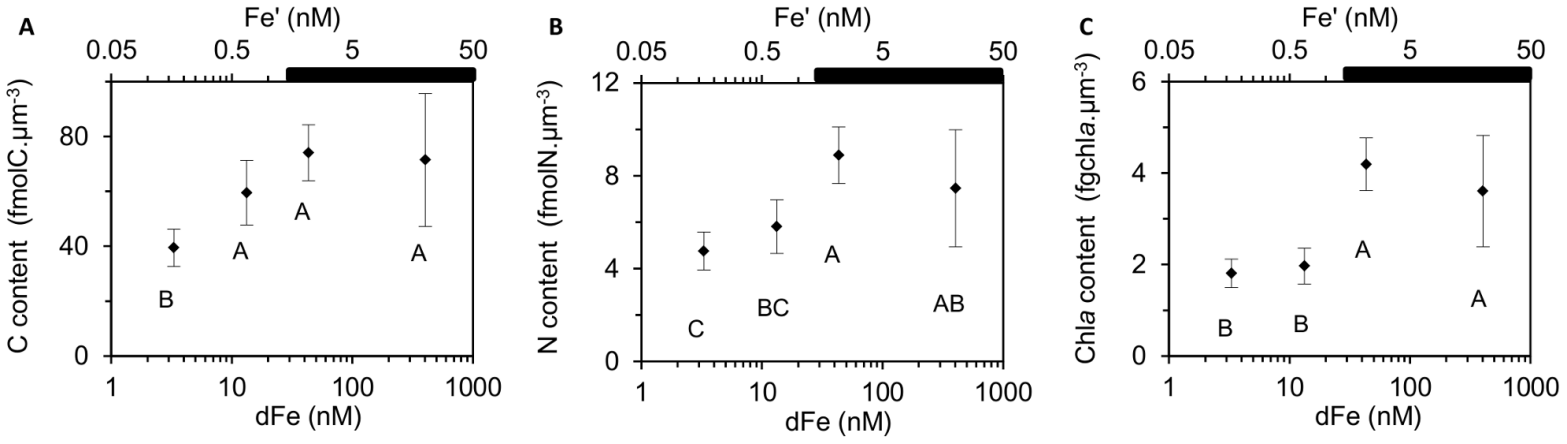
Influence of Fe availability on V-normalized contents. Mean V-normalized contents of C (A), N (B) and Chl *a* (C) of *C. watsonii* related to dFe and Fe′ concentrations, in log scale. Error bars represent standard deviation; different letters correspond to statistically different means (*p*<0.05) and the black bar indicates the region of expected Fe hydroxide precipitation.

**Figure 5:**
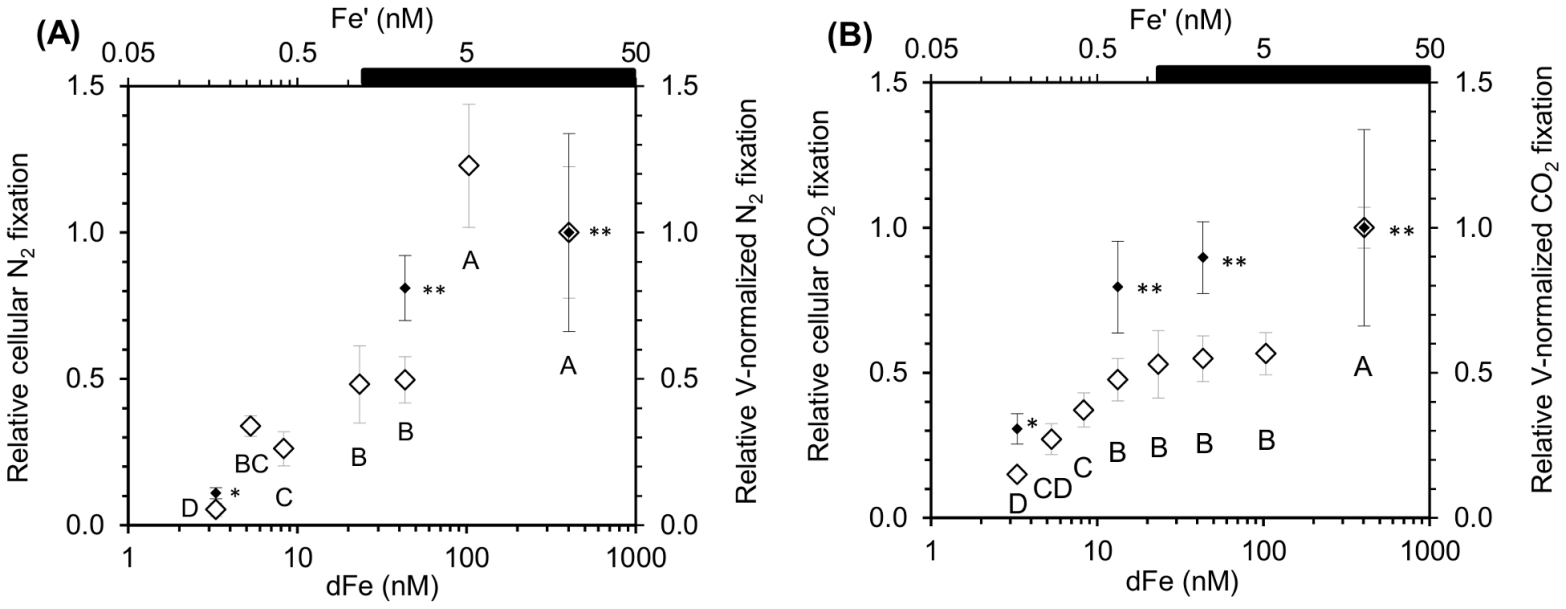
Influence of Fe availability on N_2_ and CO_2_ fixation rates of *C. watsonii*. (A) Relative N_2_ fixation rates and (B) relative CO_2_ fixation rates of *C. watsonii* related to dFe and Fe′ concentrations, in log scale. Open and filled symbols correspond to the rates per cell and V-normalized rates respectively. Error bars represent standard deviation; different letters and different numbers of star correspond to statistically different means (*p*<0.05) for cellular and V-normalized rates, respectively. The black bar indicates the region of expected Fe hydroxide precipitation.

The growth rate of *C. watsonii* was highly dependent on dFe concentrations as shown by the 2.6-fold decrease (*p*<0.05) from 0.52±0.03 d^−1^ under Fe-replete condition to 0.20±0.03 d^−1^ for the lowest dFe concentration (Figure 1). The relationship between specific growth rate and dFe concentrations fits a Monod saturation function (r^2^ = 0.92) with a maximum specific growth rate (*μ*_max_) of 0.54±0.01 d^−1^ and a half-saturation constant for growth with respect to dFe (K*_μ_*_dFe_) of 6.95±0.66 nM dFe (Figure 1). Pictures of the cells grown under Fe repletion (dFe = 403.3 nM) and severe limitation (dFe = 3.3 nM) (Figure 2A, B) illustrated the dramatic 2.2-fold decrease in the cell size with decreasing dFe concentrations, from 8.4±2.6 µm^3^ to 3.8±0.7 µm^3^ (Figure 2C). The decrease in biovolume led to a significant increase in the surface to volume (S∶V) ratio with Fe stress from 2.4±0.3 µm^−1^ (dFe = 403.3 nM) to 3.1±0.2 µm^−1^ (dFe = 3.3 nM) (Table 1). The mean cellular C and N contents in Fe-replete cultures (dFe = 403.3 nM) were 547±25 fmolC.cell^−1^ and 57±5 fmolN.cell^−1^, respectively (Figure 3A, B), resulting in a molar C∶N ratio of 9.6±0.5 (Table 1). Reducing dFe concentration to 3.3 nM induced a 3.8- and 3.3-fold decreases (*p*<0.05) in the cellular C and N contents, respectively. In all the cultures, C∶N was higher than the Redfield ratio (106∶16) and there was no correlation between the C∶N ratio and dFe concentrations (Table 1). The cellular Chl *a* content strongly declined (4.1-fold, *p*<0.05) from 28±3 fgChl*a*.cell^−1^ to 6.7±1.5 fgChl*a*.cell^−1^ over the whole range of dFe concentrations (Figure 3C) and there was no clear correlation between dFe concentrations and the Chl*a*∶C ratio (Table 1). Volume-normalized (V-normalized) C, N and Chl *a* contents decreased significantly between the 2 extreme dFe concentrations by ∼1.8-fold (*p*<0.05; Figure 4). Over the range of dFe concentrations, cellular N_2_ fixation rates declined by ∼17-fold (*p*<0.05; Figure 5A) whereas cellular CO_2_ fixation rates decreased by ∼7-fold from 29.8±2.1 fmolC.cell^−1^.h^−1^ to 4.4±0.4 fmolC.cell^−1^.h^−1^ (*p*<0.05, Figure 5B). The decrease in V-normalized N_2_ fixation rates between the two extreme dFe concentrations was much higher (9.1-fold) than that of the CO_2_ fixation rates (3.3-fold) (Figure 5, Table 1).

Under Fe-replete conditions, the growth rate, biovolume, cellular N content and C∶N ratio of *C. watsonii* reported here were in the range of published data for the WH8501 strain, and Chl*a*∶C ratio was lower than the previously published one (Table 2). Cellular C content was higher than those reported in previous studies. CO_2_ fixation rates were higher than those obtained by [50] in cultures having a considerably lower growth rate (Table 2). The growth rate obtained under Fe-replete conditions was close to that determined recently in the oligotrophic South Pacific for *C. watsonii* (0.61 d^−1^, [33]). The N_2_ fixation rates we reported are probably underestimated due to the use of the gas bubble enrichment method. Recently, it has been shown that this method may underestimate N_2_ fixation rates relative to the enriched ^15^N_2_ seawater method due to incomplete ^15^N_2_ gas bubble equilibration [51]. Based on data from these authors, the N_2_ fixation rates measured during a short incubation of 3.5 h could be underestimated at least by 70%. However, despite this potential underestimation, the relative N_2_ fixation rates should not have been affected.

**Table 2:** Comparison of growth rate, biovolume, cellular contents, elemental ratio and CO_2_ fixation rate of *C. watsonii* WH8501 cultivated under Fe-replete conditions (numbers in brackets represent standard deviation).

Growth rate	Biovolume	C content	N content	C∶N	Chl *a*∶C	C0_2_ fixation rates	
d^−1^	µm^3^	fmolC.cell^−1^	fmolN.cell^−1^	mol∶mol	µmol.mol	fmol^1^C.cell^−1^.h^−1^	Ref.
0.46			6.9–29.6	8.8 (1.5)			[46]
0.47 (0.01)	4.2–65.4						[23]
				6.9 (0.2)	83 (12)		[31]
0.54	4.2–33.5						[25]
	12–13.6			8.5*			[45]
		500	80	5.2			[47]
0.2	8.2–10.4	140–220	18–40	8.8*			[48]
0.28 (0.02)		120–260	20–35	10.5*			[49]
0.14						∼9	[50]
**0.52 (0.03)**	**8.4 (2.6)** *	**547 (25)** *	**57 (5)** *	**9.6 (0.5)** *	**58 (5)** *	**29.8 (2.1)**	**This study**

* during light period.

### 2. Influence of the degree of Fe-limitation

#### 2.1 Toward a moderate Fe deprivation (from Fe-replete condition to 43.3 nM dFe)

Our results showed two distinct responses of *C. watsonii* depending on the degree of Fe limitation (Figure S1). Under a moderate Fe limitation, corresponding to a diminution of dFe concentrations by an order of magnitude, significant decreases in cellular contents (C, N and Chl *a*) and cellular N_2_ and CO_2_ fixation rates were observed. These decreases were associated with a ∼2-fold reduction in biovolume (*p*<0.05), while the growth rate remained unchanged (*p*>0.05, Figure S1). The V-normalized C, N and Chl *a* contents as V-normalized N_2_ and CO_2_ fixation rates did not significantly change over this range of dFe concentrations (*p*>0.05, Figures 4 and 5). The decrease in the cellular contents and N_2_ and CO_2_ fixation rates can be attributed to the cell size reduction. A moderate Fe limitation induced a reduction of the cell volume which permitted to *C. watsonii* to maintain maximum C, N and Chl *a* contents as N_2_ and CO_2_-fixing activities, and hence to keep sufficient energy to sustain optimal growth rates. We suggest that the cell volume reduction of *C. watsonii* represents an adaptive strategy to decreasing Fe availability allowing to a decrease in Fe requirement and to an increase in the S∶V ratio (Table 1) which provide an advantage for the Fe uptake by increasing the diffusion-limited uptake rate relative to cell demand [39]. Increase in S∶V ratio as an adaptation to Fe limitation was previously evidenced for eukaryotic phytoplankton such as coccolithophores (*Emiliania huxleyi*) [52] and some diatoms (*Thalassosira weissflogii*, *Thalassiosira oceanica* [52] and C*haetoceros dichaeta* [53]).

Under moderate Fe-limitation conditions, estimated Fe′ concentrations exceeded the solubility limit of Fe with respect to hydroxide precipitation and thus they were not expected to vary despite the reduction of dFe concentrations. Consequently, our observations of significant physiological changes under such conditions suggest that Fe′ was probably not the only available form of Fe for *C. watsonii*. First, we can suspect that Fe from colloidal and/or precipitated amorphous Fe hydroxides is bioavailable. Recently [54] observed that both natural and cultured *Trichodesmium* are able to take up Fe from ferrihydrite (an amorphous oxidized Fe hydroxide) via cell surface adsorption and biological mediated dissolution. [55] have shown that *Trichodesmium* and the non-diazotrophic unicellular cyanobacterium *Synechoccocus* were able to take up Fe bound to recently formed organic colloids, probably involving biological reduction of colloidal Fe, leading to highly soluble Fe(II) forms [40]. Additionally, *C. watsonii* could acquire Fe from bioreduction of the Fe-EDTA complex into Fe(II) at the cell surface, as the FeEDTA complex could not be transported across the cell membrane [56]. This acquisition strategy has already been shown for the diatom *T. weissflogii*, although reduction rates are widely lower than for Fe′ [56]. Based on our results, *C. watsonii* could be able to acquire Fe from other forms than Fe′ but its Fe uptake mechanisms to be characterized.

#### 2.2 Toward a more severe Fe deprivation (from 43.3 nM to 3.3 nM dFe)

When intensifying Fe deprivation, cellular contents (C, N and Chl *a*) and cellular N_2_ and CO_2_ fixation rates continued to decrease (Figure 3, 5, S1 A to E). Over this range of Fe concentrations, the growth rate dropped significantly (*p*<0.05, Figure 1) while the cell volume remained unchanged (Figure 2C, S1F) as well as the S∶V ratio (*p*>0.05, Table 1). This indicates that the cells have reached a minimum volume, and thus a maximum S∶V ratio, around 43.3 nM dFe. As a consequence, an ∼2-fold decrease in V-normalized contents (C, N and Chl *a*) and a significant drop in V-normalized N_2_ and CO_2_ fixation rates were observed (Figure 4 and 5). Thus, under severe Fe limitation, the cellular composition and the efficiency of N_2_ and CO_2_ uptake were strongly affected and the cells were not able to produce sufficient energy to maintain an optimal growth.

In photoautotrophic cells, energy is provided as adenosine triphosphate (ATP) during respiration through the catabolism of carbohydrates produced during photosynthesis. Since a majority of redox metalloenzymes involved in this process are Fe rich proteins [30], [57], a severe reduction in bioavailable Fe induced a lower efficiency in the photosynthetic activity, as depicted by the significant decrease of V-normalized CO_2_ fixation rates and Chl *a* contents as it is the main light harvesting pigment involved in photosynthesis. Our results showed that V-normalized N_2_ fixation rates were more affected by severe Fe limitation than CO_2_ fixation rates, as illustrated by ∼7.5 and ∼3-fold decreases respectively from 43.3 to 3.3 nM dFe. The high Fe content of nitrogenase co-factors associated with the high energetic cost of biological N_2_ fixation could explain this pattern. Indeed, recent studies reported that Fe deprivation leads to a down-regulation of nitrogenase expression in both cultured and *in situ Trichodesmium* [58], [59]. This could also occur for *C. watsonii*, but it has been not yet evidenced. Additionally, N_2_ fixation is the highest energy consuming process in the cell [48], [49]. This process is fuelled by catabolism of carbohydrates accumulated during photosynthesis [60]. Thus, decreasing Fe bioavailability also affects the N_2_ fixation rates through the photosynthetic deficiency.

### 3. Comparison of the response of C. watsonii to Fe limitation with other phytoplanktonic species

To date, only two species of UCYN are available in culture: one isolated from the open ocean (*C. watsonii*, UCYN-B [20]) and one from coastal waters (*Cyanothece* WH8904, UCYN-C [61]). A study from [62] has shown no influence of Fe limitation on N_2_ fixation rates of *Cyanothece* WH8904 under a wide range of dFe concentrations (from 4 nM to 4 µM complexed with 20 µM EDTA). While the cell diameter of *C. watsonii* (2.5 µm, this study) and *Cyanothece* (∼3 µm, determined from [62]) are close under Fe-repletion, differences in Fe requirements and/or Fe acquisition between both species can be strongly suspected. The uncultivated photoheterotrophic UCYN-A do not have photosystem II of the photosynthetic apparatus [63] which contains three Fe atoms [64], and are smaller (diameter<1 µm, [23]) than *C. watsonii*, suggesting that the Fe requirements of UCYN-A are likely lower than those of *C. watsonii*. As a consequence, large differences in Fe requirements and/or Fe acquisition could exist within the UCYN community (UCYN-A, -B, -C). Bioassay experiments in the tropical North Atlantic have shown contrasted responses of UCYN activity to Fe additions. Fe addition stimulated the expression of the *nifH* gene (which encodes for the Fe component of the nitrogenase) from UCYN-B only in the western part, despite detectable dFe concentrations, while *nifH* expression from UCYN-A was not stimulated either in the Western or central part [28]. In the Eastern part, [27] observed a mesoscale variability with either UCYN-A or UCYN-B abundance stimulated by Fe addition for two close sites.

The K*_μ_*_dFe_ of *C. watsonii* was twice as low as that of *Trichodesmium erythraeum* IMS101 growing in the same conditions (13.9±3.3 nM calculated from [16, Ridame and Rochelle–Newall, unpublished data]), indicating that the growth of *C. watsonii* is less impacted by Fe limitation than that of the filamentous *Trichodesmium*. *T. erythraeum* display a much higher biovolume (∼14855 µm^3^ under Fe-replete conditions, [16]) than *C. watsonii* (8.4 µm^3^), implying a S∶V ratio of *T. erythraeum* lower (S∶V∼0.55 µm^−1^, Ridame unpublished data; [62]) than that of *C. watsonii* (S∶V = 2.4 µm^−1^). The small biovolume and large S∶V ratio provide to *C. watsonii* an advantage for Fe and other nutrients uptake. Furthermore, *C. watsonii* perform a nocturnal N_2_ fixation [45], [46] with a daily synthesis and degradation of Fe-containing proteins involved in photosynthesis and N_2_ fixation, in coordination with their utilization [30]. This Fe recycling throughout the diel cycle leads to a reduction in the cellular Fe requirement of *C. watsonii* up to 40% [30]. As *T. erythraeum* perform both photosynthesis and N_2_ fixation during the photoperiod, it probably does not employ this Fe conservation strategy to the extent used by *C. watsonii*. The Fe-rich ferredoxin, constitutively used in photosynthetic electron transport, is also an efficient electron donor for nitrogenase of *Trichodesmium* [65]. Under Fe limitation, extra Fe-free flavodoxin could be synthetized to act as an alternative electron donor instead of ferredoxin [66], as previously reported for *T. erythraeum* [67]. In contrast, no increase in flavodoxin in response to Fe stress was observed for *C. watsonii* [30]. The use of flavodoxin at night during N_2_ fixation even under Fe replete conditions appears to be an adaptation that allows *C. watsonii* to reduce cellular Fe demand [30]. These physiological characteristics reveal a lower Fe requirements of *C. watsonii* than the filamentous *T. erythraeum*, which is consistent with a higher cellular Fe∶C in *T. erythraeum* (from 69 to 87 µmol∶mol^−1^ under Fe replete conditions [14], [46]) compared to *C. watsonii* (16 µmol∶mol^−1^, [46]). Consequently, *C. watsonii* is likely better adapted to the poor Fe natural oceanic waters than *T. erythraeum*.

### 4. Oceanic relevance and biogeography of N_2_ fixers

We quantified for the first time the impact of Fe bioavailability on the growth, cell size, N_2_ fixation and photosynthesis of an open ocean UCYN, and demonstrated a physiological response depending on the degree of limitation. As photosynthesis (CO_2_ fixation) provides energy for fuelling N_2_ fixation, these two processes are tightly related. Thus, the cell response should be globally considered because nutrient limitation, such as Fe deprivation, affects cell metabolism and involves intractable feedbacks.

The quantification of the impact of Fe availability on the N_2_ fixation rates of *C. watsonii* contributes to our knowledge about the control of Fe on the N cycle in the tropical and subtropical ocean. As both growth and N_2_- and CO_2_-fixing activities of *C. watsonii* are highly Fe-dependent, its abundance and activity could be controlled by the atmospheric deposition of aeolian dust which represents the major source of new Fe to the open ocean surface waters [68]. Hence, in the tropical and subtropical oligotrophic ocean, atmospheric Fe input could enhance new production and C export to the deep ocean, through the stimulation of the growth and activity of *C. watsonii*. Based on our findings, the oceanic UCYN like *C. watsonii* could be strongly Fe limited but at a lesser extent that the filamentous diazotrophic cyanobacteria *Trichodesmium*. Thus Fe bioavailability could control the biogeography of these two N_2_ fixers. Indeed, in the South-Western Pacific [69] have observed that putative *Crocosphaera* cells were dominant at oceanic stations, while *Trichodesmium* dominated in the more Fe-rich coastal stations. They attributed this relative distribution to Fe availability since Fe is mainly supplied by coastal input in this region. Due to the lack of data about UCYN, parameterizations of N_2_ fixation in regional and global ocean models are mostly based on characteristics of *Trichodesmium* sp. (e.g. [70], [71]). Hence such biogeochemical models could be improved by the addition of a simulated UCYN, as they are expected to growth in larger niches than *Trichodesmium* regarding Fe availability. Moreover, our study supports the idea that within the UCYN community (UCYN-A, *Crocosphaera*, *Cyanothece*) Fe requirements and/or Fe acquisition may strongly vary, meaning that Fe bioavailability could partially explain variabilities in the UCYN community composition. Recent biogeochemical models focused on the biogeography of the N_2_ fixers [22], [72], [73] consider, in addition to a *Trichodesmium* analogue, a UCYN analogue parameterized with growth parameters and cellular contents derived from *Trichodesmium* and non-diazotrophic picophytoplanktonic species. Within such UCYN pool, it could be relevant to consider different types of UCYN (UCYN-A, -B and –C). In this context, the parameters obtained in our study will help to improve the parameterization of N_2_ fixation and UCYN distribution in biogeochemical models.

## Supporting Information

Figure S1 **Two distinct physiological responses of** ***C. watsonii*** **to Fe limitation.** Cellular contents of C (A), N (B), Chl *a* (C), cellular N_2_ (D) and CO_2_ fixation rates (E) and growth rates (F) related to cell volume for 4 dFe concentrations (dFe = 3.3, 13.3, 43.3 and 403.3 nM). Error bars represent standard deviation. Different numbers of stars and different letters correspond to statistically different means for the cell volume and parameters listed above.
(TIF)
