# Correction: Response of the Unicellular Diazotrophic Cyanobacterium *Crocosphaera watsonii* to Iron Limitation

**DOI:** 10.1371/journal.pone.0092655

**Published:** 2014-04-09

**Authors:** The PLOS ONE Staff

The following information is missing from the Funding section: This publication was funded by the French national IPSL project MODIF (Développement d'un MOdèle numérique du phytoplancton DIazotrophe)(Crocosphaera sp.) et de sa limitation en Fer.

In the Materials and Methods section, the second to last sentence incorrectly lists the percentage of total particulate organic carbon represented by C content associated with bacteria as 4%. The correct percentage is 2.8+/- 0.9 %

The final line of the second column of Table 2 incorrectly includes an asterisk. The correct version of Table 2 is below.

**Table 2:** Comparison of growth rate, biovolume, cellular contents, elemental ratio and CO_2_ fixation rate of *C. watsonii* WH8501 cultivated under Fe-replete conditions (numbers in brackets represent standard deviation).

Growth rate	Biovolume	C content	N content	C:N	Chl *a*:C	C0_2_ fixation rates	
d^-1^	µm^3^	fmolC.cell^-1^	fmolN.cell^-1^	mol:mol	µmol.mol	fmol^1^C.cell^-1^.h^-1^	Ref.
0.46			6.9 – 29.6	8.8 (1.5)			[46]
0.47 (0.01)	4.2 - 65.4						[23]
				6.9 (0.2)	83 (12)		[31]
0.54	4.2 -33.5						[25]
	12 - 13.6			8.5 *			[45]
		500	80	5.2			[47]
0.2	8.2 - 10.4	140 - 220	18 - 40	8.8 *			[48]
0.28 (0.02)		120-260	20-35	10.5 *			[49]
0.14						∼9	[50]
**0.52 (0.03)**	**8.4 (2.6)**	**547 (25)** *	**57 (5)** *	**9.6 (0.5)** *	**58 (5)** *	**29.8 (2.1)**	**This study**

* during light period
