# Supplementary figures and images for: Response of the Unicellular Diazotrophic Cyanobacterium Crocosphaera watsonii to Iron Limitation
Source: PLoS One. 2014 Jan 21;9(1):e86749. doi: 10.1371/journal.pone.0086749 (PMC3897776; doi:10.1371/journal.pone.0086749)

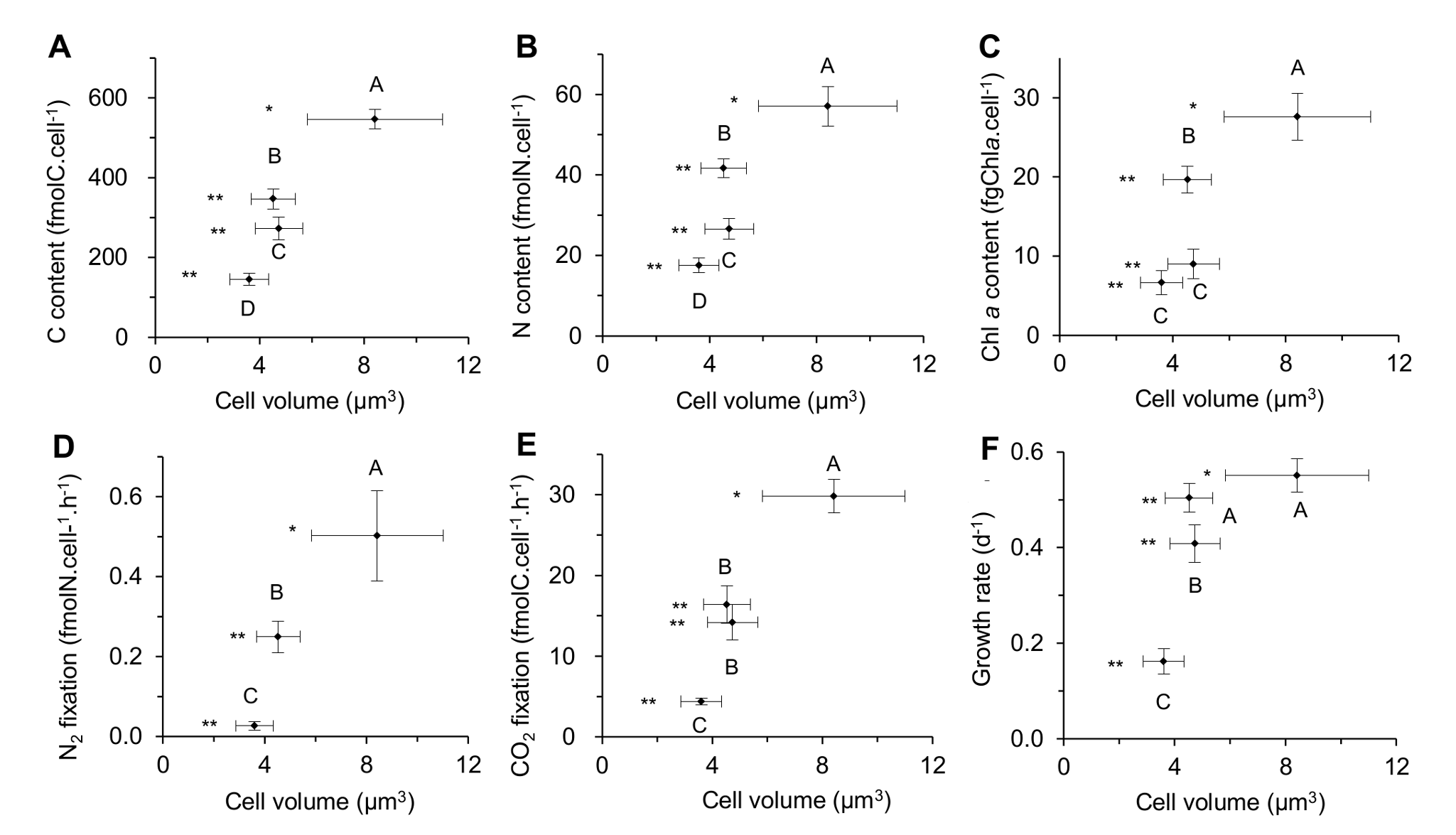

Supplement: Figure S1 — Two distinct physiological responses of C. watsonii to Fe limitation. Cellular contents of C (A), N (B), Chl a (C), cellular N2 (D) and CO2 fixation rates (E) and growth rates (F) related to cell volume for 4 dFe concentrations (dFe = 3.3, 13.3, 43.3 and 403.3 nM). Error bars represent standard deviation. Different numbers of stars and different letters correspond to statistically different means for the cell volume and parameters listed above. (TIF) [file pone.0086749.s001.tif]
